# Phenotyping Conservation Agriculture Management Effects on Ground and Aerial Remote Sensing Assessments of Maize Hybrids Performance in Zimbabwe

**DOI:** 10.3390/rs10020349

**Published:** 2018-02-24

**Authors:** Adrian Gracia-Romero, Omar Vergara-Díaz, Christian Thierfelder, Jill E. Cairns, Shawn C. Kefauver, José L. Araus

**Affiliations:** 1 Integrative Crop Ecophysiology Group, Plant Physiology Section, Faculty of Biology, University of Barcelona, 08028 Barcelona, Spain; adriangraciaromero@hotmail.com (A.G.-R.); omarvergaradiaz@gmail.com (O.V.-D.); jaraus@ub.edu (J.L.A.); 2 International Maize and Wheat Improvement Center, CIMMYT Southern Africa Regional Office, P.O. Box MP163, Harare, Zimbabwe; c.thierfelder@cgiar.org (C.T.); j.cairns@cgiar.org (J.E.C.)

**Keywords:** maize, remote sensing, UAV, RGB, multispectral, conservation agriculture, Africa

## Abstract

In the coming decades, Sub-Saharan Africa (SSA) faces challenges to sustainably increase food production while keeping pace with continued population growth. Conservation agriculture (CA) has been proposed to enhance soil health and productivity to respond to this situation. Maize is the main staple food in SSA. To increase maize yields, the selection of suitable genotypes and management practices for CA conditions has been explored using remote sensing tools. They may play a fundamental role towards overcoming the traditional limitations of data collection and processing in large scale phenotyping studies. We present the result of a study in which Red-Green-Blue (RGB) and multispectral indexes were evaluated for assessing maize performance under conventional ploughing (CP) and CA practices. Eight hybrids under different planting densities and tillage practices were tested. The measurements were conducted on seedlings at ground level (0.8 m) and from an unmanned aerial vehicle (UAV) platform (30 m), causing a platform proximity effect on the images resolution that did not have any negative impact on the performance of the indexes. Most of the calculated indexes (Green Area (GA) and Normalized Difference Vegetation Index (NDVI)) were significantly affected by tillage conditions increasing their values from CP to CA. Indexes derived from the RGB-images related to canopy greenness performed better at assessing yield differences, potentially due to the greater resolution of the RGB compared with the multispectral data, although this performance was more precise for CP than CA. The correlations of the multispectral indexes with yield were improved by applying a soil-mask derived from a NDVI threshold with the aim of corresponding pixels with vegetation. The results of this study highlight the applicability of remote sensing approaches based on RGB images to the assessment of crop performance and hybrid choice.

## 1 Introduction

Traditional practices of land preparation involve soil tillage through moldboard ploughing to soften the seedbed, ensure uniform germination, remove weed plants, and release soil nutrients through mineralization and oxidation. However, this mechanical disturbance is leading to a decline in organic matter, an increase of the loss of water by runoff, and, finally, to soil erosion [1]. Together with increasing threats of climate change, the loss of soil and its fertility is expected to become more critical for global agricultural production [2]. Over the next century, Sub-Saharan Africa (SSA) is expected to be particularly vulnerable due to the range of projected impacts: e.g., the multiple stresses and low adaptive capacity of current cropping systems, as well as population increase [3]. Maize (*Zea mays* L.) is the principal staple food crop in large parts of SSA and is usually grown in small-holder farming systems under rainfed conditions. The limited availability of inputs is a leading factor that contributes to low yields that in turn are not able to keep pace with the food demand [4]. Hence, one of the most effective pathways for adaptation is to focus on breeding new varieties and also on changing crop management [5–8].

In light of soil degradation, conservation agriculture (CA) practices have been proposed as an alternative to tillage-based agriculture in SSA as a pragmatic solution to increasing production while conserving the natural resource base [9]. CA is a set of core principles, including minimum soil disturbance, permanent soil cover, and diversified crop rotations supported by integrated soil, crop, and water management, which aims to reduce and/or revert many negative effects of conventional farming practices [10]. Besides the control of soil erosion, CA has become increasingly popular as the crop management system conserves soil moisture, reduces fossil fuel use, lowers costs and, once established, increases yield permanently [11]. Recent literature has shown the potential of CA to improve resilience against seasonal drought events and thereby reduce the risk of crop failure in SSA [8,12–16]. However, most crop cultivars currently grown under CA have been developed under conventional or full tillage conditions, and it is likely that relevant genetic adaptations of CA conditions may have been removed during previous breeding efforts. Furthermore, large scale phenotyping studies under zero-tillage conditions are missing.

For the selection of genotypes with high-performing yield components under CA, accurate phenotyping tools capable of estimating yield at early crop stages are needed. During early growth stages, environmental factors such as temperature or soil moisture have a vital influence on germination rate, seedling vigor, and, consequently, on yield [17]. However, assessing those traits usually requires destructive laboratory measurements and/or visual scoring assessments that are laborious under field conditions. They are also prone to be subjective and incur additional associated costs.

Specialized sensors have become an important component for crop monitoring, particularly for improving precision, efficiency, and throughput in phenotyping [18]. Remote sensing indexes have largely demonstrated their various applications in agriculture, including yield prediction, stress detection, and control of plant diseases under a wide range of growing and environmental conditions [19]. The classical approach has involved the use of multispectral data for the development of numerous vegetation indexes to measure biomass (e.g., Normalized Difference Vegetation Index, NDVI), water content (e.g., Water Band Index, WBI), or pigment composition (e.g., Modified Chlorophyll Absorption Ratio Index, MCARI) in yield studies. At present, the use of information derived from RGB images (using red, green, and blue color bands) acquired with conventional digital cameras represents a low-cost alternative. The images can be processed to convert RGB values into indexes based on the models of Hue-Intensity-Saturation (HIS), CIELab, and CIELuv cylindrical coordinate representations of colors [20]. Moreover, recent technological advances have led the incorporation of these sensors into aerial based platforms, enabling the simultaneous characterization of specific crop physiological traits for a larger number of plots, which may help to minimize the effect of changing environmental conditions during critical sampling moments [18,21–24].

Alternative applications of remote sensing techniques include the measurement of canopy temperature [25]. This can provide high-value information of the crop water status, since transpiration is a principal factor reducing the leaf’s temperature. The use of thermal cameras has been proposed as an easy approach in crop management, and in breeding it can replace other more laborious techniques like the use of stable isotopes, which are costly, time-consuming, and require extensive laboratory work [26]. Moreover, in case of C4 crops like maize, the usefulness of stable isotopes for breeding is questionable [27]. The possibility of applying these methodologies in CA systems could be critical in improving our knowledge and supporting the full implementation of crop phenotyping for CA in developing countries.

The aim of the present study was to evaluate the efficiency of a set of remote sensing indexes in assessing the yield differences of different maize hybrids at early growth stages under conventionally ploughed (CP) and zero-tillage (CA) conditions. Different categories of sensors were tested, including RGB cameras (placed on an aerial platform as well as at ground level), alongside multispectral and thermal cameras (both installed on the aerial platform) and an active sensor portable field spectrometer designed to measure the NDVI at ground level. Additionally, canopy temperature, leaf chlorophyll content, and dry matter isotopic composition were evaluated.

## 2 Materials and Methods

### 2.1 Site Description

**Figure 1:**
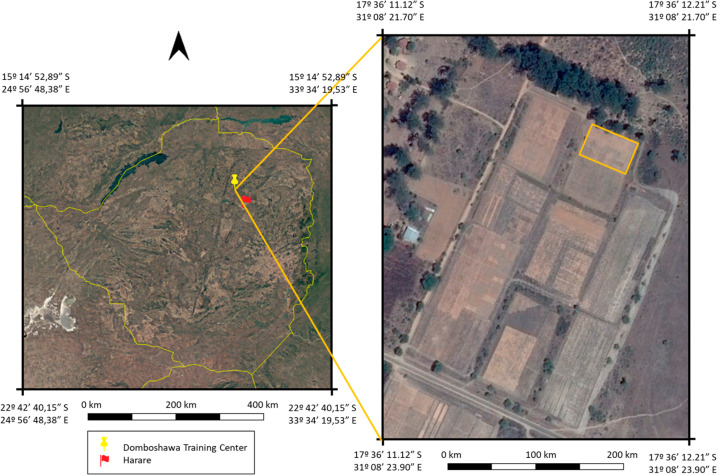
Landsat satellite (**left**) and CNES Airbus (**right**) images of the study area acquired using Google Earth Pro. The photographs are from the 31st of December 2015. The image on the left shows the location of the Domboshawa Training Center in Zimbabwe. The image on the right shows the field site.

The experiment was conducted at Domboshawa Training Centre (17*^°^*37’S, 31*^°^*10’E and 1560 m.a.s.l.), situated at the north-east of Harare (Zimbabwe), during the 2015/2016 crop season (Figure 1). This site is characterized by moderately deep Arenosols and Luvisols under FAO classification [28]. It has approximately 5% clay content and is derived from granite parent material [29]. The climate conditions correspond to the Zimbabwean agro-ecological region II [30], with generally long dry periods (April to October), in which April to July is cool and August to October is warm. This region receives an average rainfall of between 700 and 1000 mm and mean maximum daily temperatures of 32 *^°^*C during summer.

### 2.2 Plant Materials and Experimental Design

**Figure 2:**
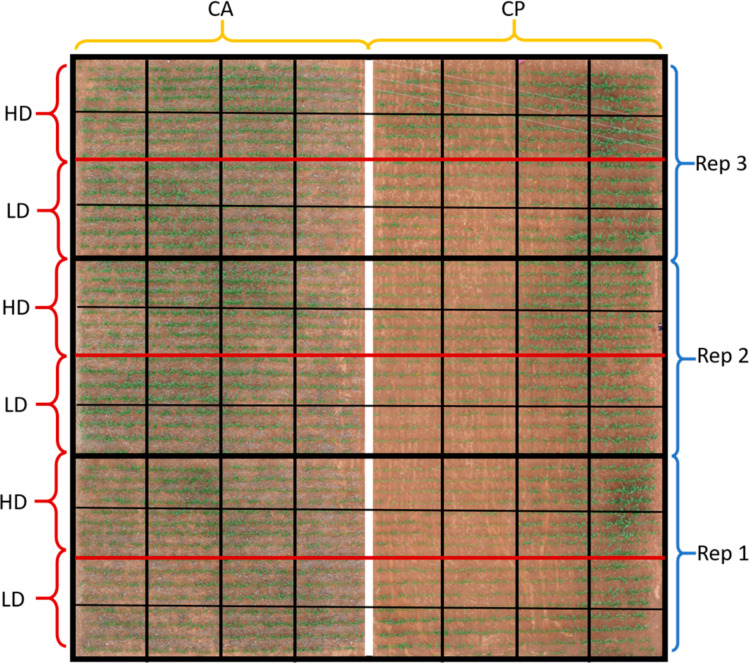
Map of the experimental design showing alternating High Density (HD) and Low Density (LD) plots per replicate, with Conservation Agriculture (CA) on the left and Conventional Ploughing (CP) on the right. Each square corresponds one plot dedicated to each of the different hybrids used. Complete details of the experimental design are explained in Section 2.2.

Seven maize drought tolerant commercial hybrids (SC621, Pan53, 30G19, Zap55, Pristine601, PGS61, and Zap61) and one drought-sensitive commercial control variety (SC513) were manually planted on 14 December 2015 in plots of 23 m^2^ (5 *×* 4.6 m) with four lines per plot. Two differential plot management regiments have been applied to the field since 2009 (Figure 2). One half was managed using no-tillage and the application of 2.5–3.0 Mg ha*^−1^* of maize stover to all the plots. Rotation, a critical component for CA, was not practiced in this trial. Weed control was done by applying a combination of 2.5 L ha*^−1^* of glyphosate, 3 L ha*^−1^* of atrazine, and1Lha*^−1^* of dual immediately after planting, if there were weeds present. The other half was conventionally ploughed and without any residue management.

Both cropping systems were fertilized twice equally with 200 kg ha*^−1^* using Compound D (7:14:7) as a basal NPK dressing and top dressed with ammonium nitrate (200 kg ha*^−1^*, 46% N) in a split application four weeks and seven weeks after seeding. Maize plants seeded at different density sub-treatments were applied: one low-planting density (44,444 plants ha*^−1^*) and one high-planting density (53,333 plants ha*^−1^*). Each sub-treatment was repeated in three replicates in which varieties were ordered in a completely randomized block design. A total of 96 plots were studied (2 agricultural practices *×* 2 density conditions *×* 8 varieties *×* 3 replicates, 24 plots per growing conditions).

### 2.3 Agronomical Traits and Proximal (Ground) Data Collection

The crop was harvested at physiological maturity. Grain yield (Mg ha*^−1^*) and total above-ground biomass (Mg ha*^−1^*) were determined as corresponding to the central 3.6 m of the two central rows of each plot (6.48 m^2^), omitting the border plants. The harvest index was calculated as grain yield as a portion of total biomass.

Proximal (ground) data was collected 45 days after sowing on 28 January 2016 when the hybrids reached the stage of 4 to 6 leaves. Leaf chlorophyll content (LCC) was measured using a Minolta SPAD-502 portable chlorophyll meter (Spectrum Technologies Inc., Plainfield, IL, USA). For each plot, five leaves were selected randomly (being the last fully expanded leave within a plant) and were measured in the middle portion of the lamina and averaged. The Normalized Difference Vegetation Index was determined at ground level using a portable spectrometer (GreenSeeker handheld crop sensor, Trimble, Sunnyvale, CA, USA) by passing the sensor over the middle of each plot at a constant height of 0.5 m above and perpendicular to the canopy (NDVI.g).

One RGB picture was taken per plot, holding the camera at 80 cm above the plant canopy in a zenithal plane and focusing near the center of each plot. The conventional digital camera used was an Olympus OM-D (Olympus, Tokyo, Japan), a digital single lens mirrorless camera with an image sensor size of 17.3 *×* 13.0 mm of 16-megapixel (MP) resolution. The images were saved in JPEG format with a resolution of 4608 *×* 3072 pixels. As the plots were too big for a single photograph, three different images samples were taken of each central row.

RGB images were subsequently analyzed using a version of the Breedpix 2.0 software (Jaume Casadesús, https://bio-protocol.org/e1488, IRTA, Lleida, Spain) adapted to JAVA8 and other RGB image analyses together integrated as freely available CIMMYT MaizeScanner plugin within FIJI (https://imagej.net/Fiji and https://github.com/George-haddad/CIMMYT). This software enables the extraction of RGB vegetation indexes in relation to different color properties [20]. Essentially, the indexes are based on either the average color of the entire image, in diverse units related to its “greenness”, or on the fraction of pixels classified as green canopy relative to the total number of pixels in the image. In HIS color space, the Hue (H) component describes the color value itself by traversing the visible spectrum in the form of an angle between 0*^°^* and 360*^°^*. Derived from the Hue portion of HIS color space, Green Area (GA) and Greener Area (GGA) analyze the proportion of green/yellow and green pixels in the image. GA is the percentage of pixels in the image in the hue range from 60*^°^* to 180*^°^*, that is, from yellow to bluish green. Meanwhile, GGA is somewhat more restrictive, because the range of hue considered by the index is from 80*^°^* to 180*^°^*, thus excluding the yellowish-green tones. Additionally, those two indexes are used to formulate the Crop Senescence Index (CSI), which provides a scaled ratio between yellow and green vegetation pixels [31], as follows:

$$CSI=\frac{\left(GA-GGA\right)}{GA}\times 100$$

In the CIELab color space model, dimension L* represents lightness, and the green to red range is expressed by the a* component, with a more positive value representing a purer red, and conversely a more negative value indicating a greener color. Meanwhile, blue to yellow is expressed by the b* component, in which the more positive the value the closer it is to a pure yellow, whereas the more negative the value the closer it is to blue. Similarly, in the CIELuv color space model, dimensions u* and v* are perceptually uniform coordinates, in which the visible spectrum starts with blue at the bottom of the space, moving through green in the upper left (mostly scaled by v*) and out to red in the upper right (mostly scaled by u*) [32].

### 2.4 Aerial Data Collection

**Figure 3:**
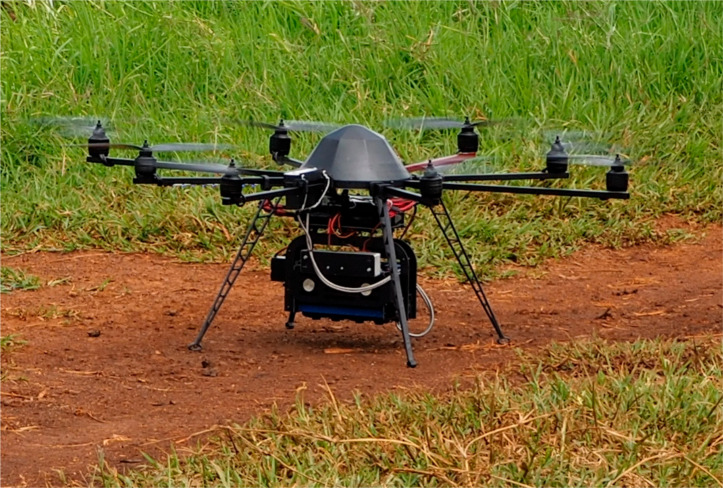
Mikrokopter OktoXL 6S12 unmanned aerial platform equipped with the micro-MCA12 Tetracam multispectral sensor, showing the placement of the Incident Light Sensor (ILS) module with white diffusor plate connected by a fiber optic cable to the top of the UAV facing upwards while the other 11 multispectral sensors are positioned on a dual axis gimbal camera platform for zenithal/nadir image capture. The RGB (Red-Green-Blue) and TIR (thermal infrared) cameras were alternately mounted on the same gimbaled platform for image capture.

Furthermore, aerial measurements were acquired during the same visit as the ground data using an unmanned aerial vehicle (UAV) (Mikrokopter OktoXL 6S12, Moormerland, Germany) flying under manual remote control at an altitude of 30 m (Figure 3). Two flights were performed; on one flight only, the RGB digital camera was mounted, and the other included both the multispectral and thermal cameras.

The RGB aerial images were obtained using a Lumix GX7 (Panasonic, Osaka, Japan) digital mirrorless camera with an image sensor size of 17.3 *×* 13.0 mm using a 20 mm lens. Images were taken at 16-MP and were saved in JPEG format, on this occasion with a resolution of 4592 *×* 3448 pixels. For the multispectral data, a Tetracam micro-MCA (Tetracam Inc., Chatsworth, CA, USA) was used. The camera consists of twelve independent image sensors and optics each with user configurable filters of center wavelengths and full-width half-max band-with (450 *±* 40, 550 *±* 10, 570 *±* 10, 670 *±* 10, 700 *±* 10, 720 *±* 10, 780 *±* 10, 780 *±* 10, 840 *±* 10, 860 *±* 10, 900 *±* 20, 950 *±* 40 nm), and one sensor dedicated to calibration (Light Incident Sensor, ILS). That sensor uses micro-filters housed in the ILS module behind a diffusor plate that corresponds to the same spectral characteristic of the 11 downwards looking sensors, thus providing an accurate band-by-band reflectance calibration in real-time. It captures 15.6-MP of image data as 12 *×* 1.3-MP images transferred to twelve separate flash memory cards. The multispectral images acquired were aligned and calibrated to reflectance using PixelWrench II version 1.2.2.2 (Tetracam, Chatsworth, CA, USA). Canopy temperature was measured using a FLIR Tau2 640 (FLIR Systems, Nashua, NH, USA) with a VOx uncooled microbolometer equipped with a TEAX Thermal Capture model (TEAX Tecnology, Wilnsdorf, Germany) for recording of full resolution thermal video (640 *×* 520 pixels at 20 frames per second). The thermal images were first exported using the TeAx ThermoViewer v1.3.12 (TeAx Technology, Wilnsdorf, Germany) to raw 16-bit TIFF format as Kelvin *×* 10,000 and converted to 32-bit temperature in Celsius using a custom batch processing macro function in FIJI [33].

To obtain an accurate orthomosaic of the pre-processed aerial images from each sensor, a 3D reconstruction was produced using Agisoft PhotoScan Professional (Agisoft LLC, St. Petersburg, Russia, www.agisoft.com)[34]. A total of 30 overlapped images were needed for each orthomosaic. Then, the procedure of cropping the plots was done using the open source image analysis platform FIJI (Fiji is Just ImageJ; http://fiji.sc/Fiji), in which regions of interest were established at each plot and then exported, taking care that exactly the same ground area was segmented for each plot across all treatments. The RGB aerial exported plots were processed the same way as the ground images as described previously in Section 2.3. For the formulation of the different multispectral indexes, as detailed in Table 1, we developed a customized FIJI macro code. This macro code enabled the calculation of the multispectral indexes through two different approaches: on the one hand, measuring the mean value of the plot image of each band (plot measurements), and on the other hand, in order to make more accurate measurements, we applied a threshold of NDVI values of 0.4–1 to focus the measurements of all the indexes on only the pixels corresponding to maize and lower NDVI values (<0.4) corresponding to bare-ground; other background image components were discarded. Therewith, percentage of vegetation cover was estimated by the inverse of the implementation of the soil mask. Finally, the thermal average temperature of the whole exported plots was also measured using FIJI.

**Table 1:** Indexes derived from the multispectral visible and near infrared bands. The wavelengths used in the formulation of the indexes are adapted slightly based on the multispectral micro-MCA Tetracam camera. * Note that for the PRI index, B550 is used instead of the original B531 by the cited reference study.

Group	Index	Equation	Wavelengths	References
Broadband Greenness	Normalized Difference Vegetation Index (NDVI)	$\frac{\left(B840-B670\right)}{\left(B840+B670\right)}$	Red, NIR	[35]
	Soil Adjusted Vegetation Index (SAVI)	$\begin{array}{c} \frac{\left(B840-B670\right)}{\left(B840+B670+L\right)}\\ \text{Intermediate vegetation, }L=0.5 \end{array}$	Red, NIR	[36]
	Optimized soil-adjusted vegetation index (OSAVI)	$\frac{\left(1+0.16\right)\cdot \left(B780-B670\right)}{\left(B780+B670+0.16\right)}$	Red, NIR	[37]
	Renormalized Difference Vegetation Index (RDVI)	$\frac{\left(B840-B670\right)}{\sqrt{\left(B840+B670\right)}}$	Red, NIR	[38]
	Enhanced Vegetation Index (EVI)	$\frac{2.5\cdot \left(B840-B670\right)}{\left(B840+\left(6\cdot B670\right)-\left(7.5\cdot R450\right)+1\right)}$	Blue, Red, NIR	[39]
Light Use Efficiency	Photochemical Reflectance Index (PRI) *	$\frac{\left(B550-B570\right)}{\left(B550-B570\right)}$	Green	[40]
Leaf Pigments	Modified Chlorophyll Absorption Ratio Index (MCARI)	$\left(B700-R670\right)-0.2\cdot (B700-B700-B550)\cdot \left(\frac{B700}{B670}\right)$	Green, Red, NIR	[41]
	Chlorophyll Content Index (CCI)	$\frac{\left(B550-B670\right)}{\left(550+B670\right)}$	Green, NIR	[42]
	Transformed Chlorophyll Absorption Ratio Index (TCARI)	Green, Red, NIR	$3\cdot \left(B700-B670\right)-0.2\cdot \left(B700-B550\right)\cdot \left(\frac{B700}{B670}\right)$	[43]
	$\frac{TCARI}{OSAVI}$		Green, Red, NIR	
	Anthocyanin Reflectance Index 2 (ARI2)	$B840\cdot \left(\frac{1}{B550}-\frac{1}{B700}\right)$	Blue, Red, NIR	[44]
	Carotenoid Reflectance Index 2 (CRI2)	$\left(\frac{1}{B550}-\frac{1}{B700}\right)$	Blue, Red	[45]
Water Content	Water Band Index (WBI)	$\left(\frac{B970}{B900}\right)$	Red, NIR	[46]

### 2.5 Carbon and Nitrogen Stable Isotope Compositions

Similar leaves were sampled for LCC, carbon, and nitrogen measurements, and were subsequently oven dried at 70 *^°^*C for 24 h and ground to a fine powder. Samples of approximately 0.7 mg of dry matter and reference materials were weighed into tin capsules, sealed, and then loaded into an elemental analyzer (Flash 1112 EA; ThermoFinnigan, Schwerte, Germany) coupled with an isotope ratio mass spectrometer (Delta C IRMS, ThermoFinnigan), operating in continuous flow mode. Measurements were carried out at the Scientific Facilities of the University of Barcelona. The ^13^C/^12^C ratios (R) of plant material were expressed in composition (δ^13^C) notation [47] as follows:

$$\delta^{13}\text{C}\left(‰\right)=[({\text{R}}_{\text{sample}}/{\text{R}}_{\text{standard}})-1]\times 1000$$

in which sample refers to plant material and standard to Pee Dee Belemmite (PDB) calcium carbonate. International isotope secondary standards of a known ^13^C/^12^C ratio (IAEA CH7, polyethylene foil, IAEA CH6 sucrose and USGS 40 l-glutamic acid) were calibrated against Vienna Pee Dee Belemnite calcium carbonate (VPDB) with an analytical precision of 0.1‰. The ^15^N/^14^N ratios of plant material were also expressed in δ notation (δ^15^N) using international secondary standards of known ^15^N/^14^N ratios (IAEA N1 and IAEA N2 ammonium sulfate and IAEA NO_3_ potassium nitrate), with analytical precision of about 0.2‰. Furthermore, total carbon and nitrogen (%) were analyzed in the same samples, and the C/N ratio was calculated.

### 2.6 Statistical Analysis

Statistical analyses were conducted using the open source software, R and RStudio 1.0.44 (R Foundation for Statistical Computing, Vienna, Austria). Data for the set of physiological traits were subjected to factorial completely randomized analyses of variance (ANOVAs) to test the effects of growing conditions on the different traits studied. A bivariate correlation procedure was used to calculate the Pearson correlation coefficients of the different remote sensing indexes against the grain yield. Multiple linear regressions were calculated via a forward stepwise method with GY as the dependent variable and the different indexes as independent parameters. The figures were also drawn using the RStudio software.

## 3 Results

### 3.1 Differences in Yield Parameters and Conventional Phenotyping Measurements within Growing Conditions and Genotypes

Means of yield, biomass, and traits informing on the water and nitrogen status of the crop are presented in Table 2. Grain yield was significantly greater under CA conditions (*p<* 0.0001), by almost 20% relative to the CP. Harvest index was also significantly higher, but no differences were found for biomass between the two treatments. Concerning planting density, it did not affect any yield parameter. Even so, the highest-yielding conditions were recorded at high-density CA plots (3.07 Mg ha*^−1^* on average), and the least on low-density CP plots (2.29 Mg ha*^−1^*). The genotypic variability for grain yield (Table 3) was only significant under CA (*p<* 0.001) at high density conditions (*p<* 0.01).

**Table 2:** Effect of the tillage conditions and planting density conditions on the yield parameters (GY, Biomass and Harvest Index), leaf chlorophyll content (LCC), leaf carbon and nitrogen concentration (C and N), leaf C/N ratio and the stable carbon (δ^13^C), and nitrogen (δ^15^N) isotopic composition. Values are means *±* standard error. Level of significance (*p*-value): *, *p* < 0.05; ***, *p* < 0.001. Treatments: CA, conservation agriculture; CP, conventional agriculture; LD, low density; HD, high density.

	GY	Biomass	Harvest Index	SPAD	Temperature	C	δ^13^C	N	δ15N	C/N
	(Mg ha^−1^)	(Mg ha^−1^)			(°C)	(%)	(‰)	(%)	(‰)	
Treatment										
*CA*	2.99 ± 0.10	2.66 ± 0.11	0.49 ± 0.01	42.71 ± 0.38	25.02 ± 0.10	45.8 ± 0.50	−11.98 ± 0.03	3.77 ± 0.04	0.52 ± 0.08	12.14 ± 0.06
*CP*	2.42 ± 0.14	2.50 ± 0.14	0.44 ± 0.01	42.24 ± 0.38	24.64 ± 0.15	44.64 ± 0.49	−11.93 ± 0.03	3.69 ± 0.04	0.59 ± 0.07	12.11 ± 0.05
*p*-value	0.000***	0.351	0.000***	0.395	0.017*	0.097	0.287	0.161	0.518	0.673
Density										
*LD*	2.61 ± 0.12	2.45 ± 0.12	0.47 ± 0.01	42.37 ± 0.36	24.59 ± 0.12	45.32 ± 0.58	−11.95 ± 0.03	3.74 ± 0.05	0.51 ± 0.07	12.13 ± 0.05
*HD*	2.81 ± 0.13	2.71 ± 0.13	0.46 ± 0.01	42.45 ± 0.40	25.14 ± 0.11	45.08 ± 0.41	−11.96 ± 0.03	3.72 ± 0.04	0.59 ± 0.08	12.12 ± 0.06
*p*-value	0.230	0.145	0.820	0.712	0.000***	0.724	0.922	0.760	0.453	0.988
Combinations										
CA * LD	2.92 ± 0.13	2.59 ± 0.17	0.49 ± 0.01	42.61 ± 0.56	24.80 ± 0.13	46.64 ± 0.48	−11.97 ± 0.04	3.83 ± 0.04	0.53 ± 0.11	12.18 ± 0.07
CA * HD	3.07 ± 0.14	2.73 ± 0.15	0.49 ± 0.01	42.81 ± 0.52	24.32 ± 0.21	44.99 ± 0.85	−11.98 ± 0.04	3.72 ± 0.07	0.50 ± 0.12	12.10 ± 0.09
CP * LD	2.29 ± 0.19	2.31 ± 0.18	0.44 ± 0.01	42.14 ± 0.47	25.25 ± 0.13	44.1 ± 0.97	−11.94 ± 0.04	3.66 ± 0.08	0.49 ± 0.09	12.07 ± 0.08
CP * HD	2.55 ± 0.20	2.69 ± 0.21	0.44 ± 0.01	42.34 ± 0.61	24.99 ± 0.17	45.18 ± 0.08	−11.93 ± 0.05	3.72 ± 0.02	0.68 ± 0.11	12.14 ± 0.07
*p*-value	0.751	0.484	0.931	0.999	0.489	0.051	0.812	0.146	0.312	0.353

**Table 3:** Genotypic yield variability of the eight maize hybrids for each growing condition. Values are means *±* standard error. Level of significance: **, *p<* 0.01. Treatments: CA, conservation agriculture; CP, conventional agriculture; LD, low density; HD, high density.

	CA	CP	LD	HD
SC513	2.50 ± 0.18	1.52 ± 0.19	2.12 ± 0.34	1.90 ± 0.20
SC621	2.32 ± 0.18	2.60 ± 0.39	2.54 ± 0.19	2.38 ± 0.40
PAN53	3.26 ± 0.13	2.71 ± 0.36	2.82 ± 0.23	3.16 ± 0.33
30G19	2.52 ± 0.15	2.12 ± 0.34	2.22 ± 0.26	2.42 ± 0.29
Zap55	3.72 ± 0.18	3.03 ± 0.39	3.23 ± 0.36	3.52 ± 0.31
Pristine	601	3.16 ± 0.26	2.02 ± 0.37	2.19 ± 0.39	2.98 ± 0.34
PGS61	3.23 ± 0.25	2.61 ± 0.29	2.76 ± 0.25	3.07 ± 0.34
Zap61	3.24 ± 0.29	2.74 ± 0.53	2.95 ± 0.51	3.03 ± 0.35
*p*-value	0.001**	0.147	0.155	0.007**

ANOVA analysis showed no significant differences in LCC between management practices or density levels. Meanwhile, canopy temperature showed a significant increase in plots grown under CA (*p <* 0.05) and when the plant density was high (*p <* 0.001), although the increment was small. Finally, neither the percentage of carbon nor nitrogen nor their isotopic signatures showed significant changes across the different growing conditions.

### 3.2 The Effect of Conservation and Conventional Agricultural Practices and the Sensor Altitude on Vegetation Indexes

The mean of the RGB and multispectral indexes for the ground and aerial images at each growing condition are shown in Tables 4 and 5. There were no significant differences across the indexes reported due to changes of planting density; mean data of low and high densities is not presented. The differential tillage practices, however, affected all the RGB indexes for both sensor levels (Table 4). Hue increased greatly from the CP to the CA (ground 20.22%/aerial 21.89%), and its derived indexes GA and GGA also showed a similar rise of their values. Otherwise, the indexes derived from the CIE-Lab and CIE-Luv reduced their values, particularly a* and u*. The height level from which the indexes were measured (either from ground or the aerial platform) affected their values at both agricultural practices conditions, except for the Saturation (Supplementary Table S1). Differences were highly evidenced in the Hue and GGA values, which decreased greatly when they were calculated from the aerial images in comparison to the ground images. Even considering those differences, the correlations between the measurements from both platforms were very strong in general, only showing low relationship coefficients with Intensity, Lightness, and the v* index. Moreover, the correlations were higher at CP conditions in comparison with CA.

**Table 4:** Effect of tillage practices and placement of the sensors (ground versus aerial) on the RGB indexes. These indices are defined in Materials and Methods. Values are means *±* standard error. Level of significance (*p*-value): ***, *p<* 0.001. Treatments: CA, conservation agriculture; CP, conventional agriculture.

		Intensity	Hue	Saturation	Lightness	a*	b*	u*	v*	GA	GGA	CSI
Ground	*CA*	0.37 ± 0.00	48.04 ± 0.97	0.19 ± 0.00	44.01 ± 0.23	−4.44 ± 0.28	20.14 ± 0.35	3.59 ± 0.42	22.91 ± 0.36	0.26 ± 0.01	0.24 ± 0.01	8.83 ± 0.60
	*CP*	0.36 ± 0.00	39.96 ± 0.91	0.25 ± 0.00	42.81 ± 0.19	−1.27 ± 0.38	23.13 ± 0.24	9.12 ± 0.57	24.79 ± 0.21	0.19 ± 0.01	0.18 ± 0.01	6.48 ± 0.46
	*p*-value	0.000***	0.000***	0.000***	0.000***	0.000***	0.000***	0.000***	0.000***	0.000***	0.000***	0.002***
Aerial	CA	0.49 ± 0.00	38.25 ± 0.75	0.19 ± 0.00	54.96 ± 0.35	−0.54 ± 0.32	24.21 ± 0.13	11.67 ± 0.54	28.10 ± 0.14	0.22 ± 0.01	0.15 ± 0.01	32.87 ± 1.19
	CP	0.49 ± 0.00	31.38 ± 0.49	0.25 ± 0.00	55.25 ± 0.48	4.99 ± 0.39	28.77 ± 0.27	22.2 ± 0.76	31.34 ± 0.27	0.16 ± 0.01	0.10 ± 0.01	42.43 ± 2.41
	p-value	0.000***	0.000***	0.000***	0.000***	0.000***	0.000***	0.000***	0.000***	0.000***	0.000***	0.000***

Excluding WBI and TCARI and the vegetation measurement of SAVI, RDVI, and MCARI, all the vegetation indexes based on multispectral data could distinguish the plots where different agricultural practices were applied (Table 5). The indexes considered as indicators of green biomass, like NDVI (both ground and aerial), SAVI, or OSAVI, exhibited a large increase (*p<* 0.050) in their values at the CA plots in comparison with CP. Contrary to this, CA showed a significant decrease (*p <* 0.001) in the percentage of vegetation cover (CA 66.25%/CP 72.30%). Indexes more related to leaf pigments, like PRI or MCARI, were also significantly higher under CA compared with CP, but it was much less pronounced. Conversely, the stress index ARI2 decreased significantly (*p<* 0.0001) from the CA to the CP. The application of soil mask, to direct the multispectral measurements to only the vegetation, resulted in significant variation in vegetation index values. The mean values of the green biomass and the vegetation pigments indexes both increased regardless of tillage conditions; the differences between the two growing conditions was much less severe than when they were measured at the whole plot level. In comparison with measurements made by GreenSeeker, raw NDVI measurements taken with the UAV were slightly lower, while those where NDVI was calculated through the mask were higher. The relationships between the measurements of NDVI at both platform levels were robust, but the correlation using the masked values were stronger (Supplementary Table S1). Moreover, these correlations were higher under CP conditions as compared with CA.

**Table 5:** Effect of tillage practices (CA versus CP) and the application of the soil mask (plot measurements versus vegetation measurements) on the multispectral indexes. These indexes are defined in Materials and Methods. Values are means *±* standard error. Level of significance (*p*-value): *, *p<* 0.05; **, *p<* 0.01; ***, *p<* 0.001. Treatments: CA, conservation agriculture; CP, conventional agriculture.

		Vegetation	NDVI.g	NDVI	SAVI	OSAVI	RDVI	EVI	PRI	MCARI	CCI
Plot	*CA*	66.25 ± 0.93	0.55 ± 0.01	0.42 ± 0.01	0.27 ± 0.01	0.34 ± 0.01	4.19 ± 0.10	0.54 ± 0.01	0.16 ± 0.00	19.54 ± 0.47	0.08 ± 0.01
	*CP*	72.30 ± 1.04	0.48 ± 0.01	0.38 ± 0.01	0.25 ± 0.01	0.31 ± 0.01	3.85 ± 0.11	0.39 ± 0.01	0.13 ± 0.00	16.89 ± 0.38	−0.01 ± 0.01
	*p*-value	0.000***	0.000***	0.006**	0.031*	0.013*	0.026*	0.000***	0.000***	0.000***	0.000***
Vegetation	*CA*	0.66 ± 0.01	0.45 ± 0.01	0.55 ± 0.01	6.85 ± 0.11	0.88 ± 0.02	0.21 ± 0.00	32.16 ± 0.66	0.27 ± 0.01
	*CP*	0.62 ± 0.01	0.44 ± 0.01	0.53 ± 0.01	6.68 ± 0.11	0.74 ± 0.01	0.19 ± 0.00	31.74 ± 0.61	0.20 ± 0.01
	*p*-value	0.001**	0.343	0.049*	0.263	0.000***	0.000***	0.624	0.000***
*TCARI*	*TCARI/OSAVI*	*ARI2*	*CRI2*	*WBI*					
Plot	*CA*	36.40 ± 0.67	0.42 ± 0.01	0.47 ± 0.01	0.01 ± 0.00	0.94 ± 0.01					
	*CP*	35.68 ± 0.74	0.47 ± 0.02	0.73 ± 0.01	0.01 ± 0.00	0.93 ± 0.00					
	*p*-value	0.461	0.030*	0.000***	0.000***	0.558					
Vegetation	*CA*	46.75 ± 0.82	0.33 ± 0.01	0.23 ± 0.02	0.00 ± 0.00	1.01 ± 0.01					
	*CP*	50.15 ± 1.10	0.38 ± 0.01	0.51 ± 0.02	0.01 ± 0.00	0.99 ± 0.00					
	*p*-value	0.012*	0.001**	0.000***	0.000***	0.06					

### 3.3 Performance of Remote Sensing Index as Predictors of Grain Yield

To test the accuracy of remote sensing indexes for assessing differences in yield, correlations are presented for grain yield against indexes. RGB indexes measured on the CP plots, regardless of height level of image taking; Hue and its derived indexes were the ones that correlated best with grain yield. Indeed, nearly all aerial indexes worked very similarly as the ground indexes, showing almost identical correlation coefficients (Table 6). Only b* and v* correlations varied considerably, going from low (ground) to extremely strong (aerial) correlations. On the other hand, even though these indexes performed much better under the conventional practices, some significant and strong correlations could be found under CA. For both ground and aerial levels, those indexes related to the greenness of the vegetation color, as Hue, GA, GGA, a*, and u* were best correlated to yield.

**Table 6:** Regression coefficients of the relationships between the RGB-indices, measured at ground and aerial levels, with grain yield. These indices are defined at section Material and Methods. Level of significance (*p*-value): *, *p* < 0.05; **, *p* < 0.01; ***, *p* < 0.001. Treatments: CA, conservation agriculture; CP, conventional agriculture; LD, low density; HD, high density.

		Intensity	Hue	Saturation	Lightness	a*	b*	u*	v*	GA	GGA
*Ground measurements*	*Tillage*										
	*CA*	−0.065	0.484***	−0.317*	−0.021	−0.509*	−0.226	−0.544***	−0.137	0.487***	0.507***
	*CP*	0.503***	0.741***	−0.478***	0.568***	−0.742***	−0.206	−0.717***	0.157	0.777***	0.783***
	*Planting density*										
	*LD*	0.342*	0.597***	−0.483***	0.366*	−0.633***	−0.350*	−0.623***	−0.152	0.660***	0.662***
	*HD*	0.361*	0.754***	−0.509***	0.410**	−0.785***	−0.326*	−0.771***	−0.100	0.767***	0.767***
	*Combinations*										
	CA * LD	−0.054	0.422*	−0.386	−0.032	−0.498*	−0.310	−0.539**	−0.213	0.511*	0.513*
	CA * HD	−0.054	0.580**	−0.247	0.020	−0.570**	−0.138	−0.585**	−0.052	0.493*	0.528**
	CP * LD	0.419*	0.622	−0.304	0.456*	−0.596**	−0.032	−0.567**	0.218	0.627**	0.636***
	CP * HD	0.565**	0.827***	−0.594**	0.671***	−0.850***	−0.318	0.830***	0.176	0.894***	0.898***
*Aerial mesurements*	*Tillage*										
	*CA*	−0.393**	0.548***	−0.149	−0.363*	−0.567***	−0.265	−0.554*	−0.145	0.562***	0.561***
	*CP*	−0.776***	0.754***	−0.719***	−0.777***	−0.796***	−0.818***	−0.812***	−0.794***	0.784***	0.798***
	*Planting density*										
	*LD*	−0.555***	0.614***	−0.495***	−0.565***	−0.651***	−0.606***	−0.658***	−0.617***	0.653***	0.664***
	*HD*	−0.627***	0.684***	−0.440**	−0.631***	−0.739***	−0.667***	−0.751***	−0.697***	0.776***	0.786***
	*Combinations*										
	CA * LD	−0.468*	0.582**	−0.292	−0.444*	−0.600**	−0.395	−0.594**	−0.296	0.578**	0.572**
	CA * HD	−0.360	0.519**	−0.105	−0.331	−0.544**	−0.262	−0.533**	−0.116	0.545**	0.557**
	CP * LD	−0.606**	0.577**	−0.649***	−0.610**	−0.654***	−0.694***	−0.674***	−0.662***	−0.662**	0.633***
	CP * HD	−0.907***	0.881***	−0.793***	−0.906***	−0.900***	−0.920***	−0.913***	−0.905***	0.916***	0.920***

Likewise, as happened with the RGB, multispectral indexes performed better when assessing yield at the conventional tillage conditions (Table 7). However, in this case CA conditions showed much lower correlations in comparison with the RGB derived indexes. Under CP conditions, NDVI and its optimized indexes (SAVI, OSAVI and RDVI) were very closely related to GY, but the strongest correlation was found with the combination of two indexes, TCARI/OSAVI (R = *−*0.779, *p<* 0.0001). For both tillage conditions, CA and CP, the application of the soil mask helped to improve the correlation between indexes related to the light use efficiency (PRI) and to the leaf pigments (TCARI) and grain yield.

**Table 7:** Regression coefficients of the relationships between the multispectral-indices, measured at ground and aerial levels, with grain yield. These indices are defined at section Material and Methods. Level of significance (*p*-value): *, *p* < 0.05; **, *p* < 0.01; ***, *p* < 0.001. Treatments: CA, conservation agriculture; CP, conventional agriculture; LD, low density; HD, high density.

		Vegetation area	NDVI.g	NDVI	SAVI	OSAVI	RDVI	EVI	PRI	MCARI	CCI	TCARI	TCARI/OSAVI	ARI2	CRI2	WBI
*Plot measurements*	*Tillage*															
	*CA*	−0.386**	0.490**	0.361*	0.389**	0.379**	0.390**	0.368*	0.435**	0.215	0.355*	0.094	−0.334*	−0.321	−0.299*	0.116
	*CP*	−0.727***	0.812***	0.751***	0.729***	0.747***	0.734***	0.676***	0.697***	0.105	0.710***	−0.454**	−0.785	−0.479	0.066	0.412**
	*Planting density*															
	*LD*	−0.592***	0.740**	0.577***	0.477***	0.532***	0.493***	0.488***	0.641***	0.127	0.610***	−0.253	−0.671***	−0.549***	−0.372*	0.269
	*HD*	−0.737***	0.795**	0.750***	0.748***	0.753***	0.749***	0.714***	0.713***	0.495***	0.731*	−0.285	−0.755***	−0.500***	−0.26	0.364*
	Combinations															
	CA * LD	−0.38	0.464*	0.298	0.281	0.289	0.285	0.251	0.398	0.108	0.327	0.006	−0.379	−0.483*	−0.359	0.065
	CA * HD	−0.393	0.526*	0.402	0.481*	0.444*	0.475*	0.528*	0.473*	0.415	0.397*	0.143	−0.367	−0.263	−0.19	0.284
	CP * LD	−0.602**	0.793**	0.630***	0.582**	0.614**	0.590**	0.490**	0.617**	−0.079	0.589**	−0.445*	−0.726***	−0.478*	0.033	0.622**
	CP * HD	−0.850***	0.850***	0.860***	0.847***	0.858***	0.849***	0.830*	0.780***	0.363	0.818***	−0.587**	−0.869	−0.488*	0.141	0.443*
*Vegetation measurements*	*Tillage*															
	*CA*	0.383**	0.390**	0.395**	0.396**	0.29	0.456**	0.13	0.344*	0.072	−0.234	−0.38	−0.390**	0.107
	*CP*	0.767***	0.664***	0.731***	0.673***	0.507***	0.729***	−0.323*	0.738	−0.641***	−0.785***	−0.695	−0.643***	0.445**
	Planting density															
	*LD*	0.617***	0.358*	0.489***	0.384**	0.333*	0.620***	−0.234	0.599***	−0.516***	−0.704***	−0.608	−0.533***	0.238
	*HD*	0.791***	0.717***	0.768***	0.723***	0.640***	0.801***	−0.062	0.803***	−0.562***	−0.752***	−0.74	−0.740***	0.503***
	*Combinations*															
	CA * LD	0.324	0.257	0.273	0.254	0.175	0.328	−0.021	0.245	−0.097	−0.404	−0.307	−0.248	0.011
	CA * HD	0.468*	0.579**	0.543*	0.577**	0.388	0.614**	0.222	0.541**	0.131	−0.238	−0.609	−0.658***	0.344
	CP * LD	0.650***	0.489***	0.588***	0.505*	0.245	0.613**	−0.402	0.596**	−0.610**	−0.732***	−0.614	−0.517**	0.444*
	CP * HD	0.866***	0.788***	0.837***	0.793***	0.702**	0.828***	−0.374	0.854***	−0.787***	−0.874***	−0.763	−0.738***	0.582**

In terms of density conditions, both RGB and multispectral indexes worked slightly better with higher plant densities compared to the low-density conditions. Moreover, a deeper analysis of the results showed that the best conditions in which to correlate the remote sensing indexes was the combination of high density planting at the CP plots. Under these conditions, most of the R^2^ coefficients were near or even higher than 0.800.

The ability of remote sensing indexes assessing grain yield was further tested by multivariate linear models (Table 8). All the equations presented were obtained using four or less indexes, all of them measured from the UAV. The best predictive equation was achieved using CP data, explaining 75.6% of the variation in yield. Besides this, the equation derived from the CA plots could only explain 35.1% of yield variation. Equations derived from the high-planting density conditions predicted yields more efficiently than low density conditions (65.4 and 52.6%, respectively).

**Table 8:** Multilinear regression (stepwise) of grain yield (GY) as dependent variable and the remote sensing traits (RGB and multispectral vegetation indexes) measured from the unmanned aerial vehicle as independent variables. These indexes are defined at section Material and Methods. R^2^, determination coefficient; RSE, Residual Standard Error.

	Equation	R2	RSE	p-Value
Conservation	GY = −4.37 + 0.11·Lightness + 9.23·GA − 0.04·MCARI		0.351	0.521	0.000
Portion of variance	Lightness	0.237			
	GA	0.164			
	MCARI	0.159			
Conventional	GY = 16.56 −0.36·b* − 16.28·SAVI + 2.06·RDVI − 0.02·TCARI/OSAVI		0.757	0.491	0.000
Portion of variance	b*	0.237			
	SAVI	0.164			
	RDVI	0.159			
TCARI/OSAVI	0.195			
Low density	GY = 5.36 − 0.26·b* + 10.21·OSAVI − 13.99·CCI		0.516	0.609	0.000
Portion of variance	b*	0.203			
	OSAVI	0.166			
	CCI	0.147			
High density	GY = 0.517 + 6.44·GGA + 3.14·OSAVI	0.654	0.531	0.000
Portion of variance	GGA	0.353			
	OSAVI	0.300			

## 4 Discussion

### 4.1 Implications of Growing Conditions on Yield Parameters

Conservation agriculture practices have been proposed as potential systems to increase crop yield, [1,2,48,49]). As can be seen in our results, CA plots out-yielded conventional agriculture. Positive responses to CA are principally the result of the interacting effect of soil characteristics and climate [1]. One of these benefits is attributed to the water-harvesting effects of minimum-tillage practices [50,51]. Underwater limited conditions increased soil moisture, leading to comparably higher yields under CA [9]. We did not report any water data in our study but indirectly derived these through sensors that measure related parameters. We attributed significant differences in plot temperature due to residue cover under CA. It is very likely that the residue cover, even though it helps to maintain the humidity of the top-layers of the soil, dries faster than the soil and thus presents a higher temperature than both soil and vegetation. Also, better water availability of CA should have been reflected in a significant decrease in the carbon isotopic composition of the leaves of this C4 species in comparison with CP agriculture plots due to a decrease of the leakiness of the bundle-sheath cells or by an increase of the C_i_/C_c_ as a consequence of an increase of the photosynthetic capacity [27]. Therefore, due to the fact that the trial has been running under CA since 2009, yield improvements were likely not related due to increased soil moisture or decreased temperature but due to a long-term improvement in soil health and fertility.

Since crop management has led to a considerable increase in yield, changes in genotype may be an option to make use of the enhanced yield potential provided by this environmental factor. While the literature related to CA is mostly focused on crop management, the study of the genetic crop adaptation is limited and usually concludes that the interaction between the tillage practice and the genotype is absent [7,52–54]. Crops have been grown on conventional tillage for many years and genes governing the adaptation to CA either have been lost over time through untargeted selection or have become redundant [55]. However, the varieties used in this experiment only showed significant differences in yield under CA, not under conventional agriculture management (CP). This may suggest the existence of some traits linked to tillage with a direct effect on improving yield. Herrera et al. (2013) [56] conclude that traits associated with emergence (early vigor) and resistance to diseases may increase genotype performance under CA. Thus, these results reinforce the need to further evaluate genotypic performance of varieties developed and selected in CP and test them under no-tillage conditions.

### 4.2 Comparative Performance of the Vegetation Indexes at Determining Differences in Grain Yield under CP and CA Conditions

RGB imaging and processing have become a major tool for phenotyping, and its ability to determine plant performance in terms of biomass and yield has been demonstrated again in this study. The indexes that performed better in assessing differences in yield were the ones more related to canopy greenness (such as a* or GGA) and thus to vegetation cover [20]. Therefore, elevated values of these indexes, driven by higher biomass levels, help to anticipate higher yields even at early growing stages [57]. Just like RGB, the multispectral indexes that are more sensitive to the green biomass (e.g., NDVI) and its reformulations such as the SAVI, OSAVI, and RDVI were the best correlated with GY. Those indexes contain information from the red reflectance region [35,37,38], which increases with a reduction of the biomass density, making them ideal for identifying differences in vigor at early growing stages.

Other multispectral indexes that worked well in assessing differences in GY were PRI and TCARI/OSAVI. The Photochemical Reflectance Index (PRI) is a spectral index increasingly used as an indicator of photosynthetic efficiency [58], because it is closely related to ΔF/Fm’ [59,60]. Low PRI values reflect a lower light use efficiency of PSII that will finally be translated in a yield loss. Meanwhile, the Transformed Chlorophyll Absorption in Reflectance Index (TCARI), based on the Modified Chlorophyll Absorption in Reflectance Index (MCARI), is a depth measure of the chlorophyll absorption at 670 nm relative to the reflectance at 550 and 700 nm [43]. This pigment index did not correlate with yield until the background was corrected and normalized with the OSAVI. The TCARI/OSAVI ratio is an index very sensitive to changes in chlorophyll content but very resistant to the variations in LAI at the same time [43].

**Figure 4:**
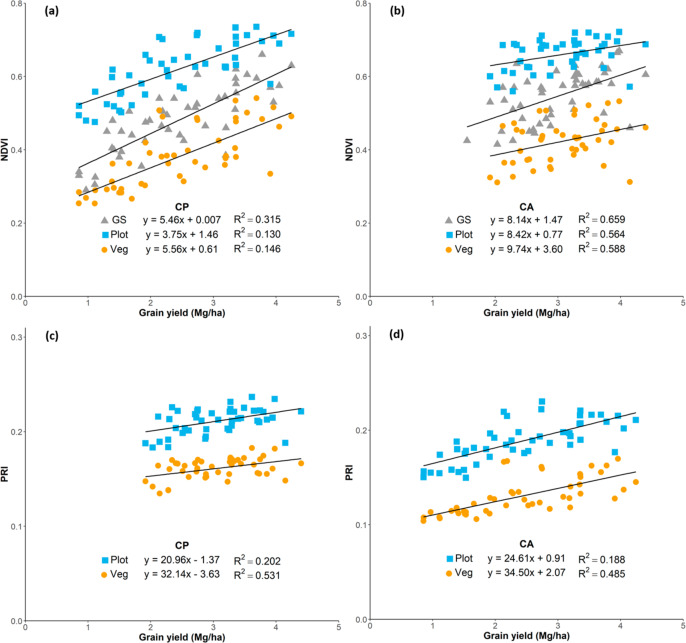
Relationship between grain yield with the Normalized Difference Vegetation Index (NDVI), measured with the GreenSeeker and calculated from the aerial multispectral images (**a**,**b**) and with the Photochemical Reflectance Index (PRI), measured from the aerial multispectral images (**c**,**d**).

**Figure 5:**
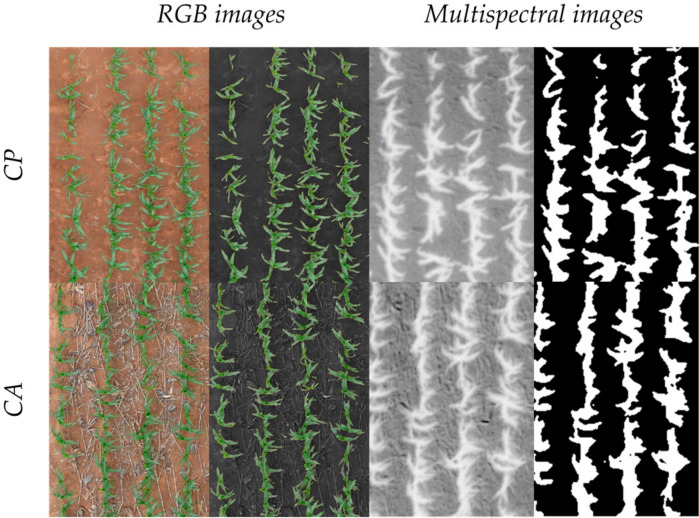
Examples of the differences in the vegetation area identification with the RGB and multispectral images with at the conservation (ca) and conventional (CP) plots.

Although significant results were obtained, these indexes did not perform equally when assessing yield differences within the different tillage growing conditions. The strengths of the indexes’ (both RGB and multispectral) correlations against yield were much lower in CA compared with CP. The reason for this is assumed to be the added noise derived from the crop residue coverage of the soil. According to the FAO definition, the soil surface has to be covered at least by 30% to qualify as CA in principle [61], which may have influenced remote sensing readings under CA. Due to this fundamental difference between CA and CP, it is difficult to segregate the biomass from the plant and residue cover. The application of an NDVI mask on the multispectral images effectively reduced background reflectance and increased their correlations statistically, although the improvements were minor (Figure 4). Indexes linked to the pigment content such as the CCI, ARI2, and CRI2 benefited most from the use of a soil mask. As can be seen in Figure 5, even with its distinct color, the CA background influenced the images mildly and supported the assessment of vegetation area, particularly in RGB images that are based on the portion of green pixels of the image.

Meanwhile, the use of the near-infrared (NIR) region by some spectral indexes, which greatly decreases its reflectance over soil, helps to increase the sensibility to the canopy cover [62]. Despite these appreciations, the RGB-based indexes GA and GGA outperformed NDVI and the rest of spectral indexes at predicting GY under CA conditions. The far higher resolution of the RGB compared with the multispectral images may be the critical factor here when working from an aerial platform (Figure 2)[33,57].

### 4.3 Platform Proximity Effects on the Performance of the Vegetation Indexes Assessing Grain Yield Differences

The possibility to incorporate remote sensing methodologies onto unmanned aerial based platforms enables the characterization of a larger number of plots in much less time, helping to minimize the effect of the changing environmental conditions during the sampling [18]. Moreover, aerial photographs facilitate the coverage of the whole plot (which usually is not possible for the images taken at ground level, particularly with tall crops such as maize). Yet, the altitude of the sensor from the canopy of the crop has a negative effect on the resolution of the images when using cameras with the same resolution (16-megapixels with both airborne and ground). While the RGB images collected from the UAV only reached a resolution of 825 *×* 1210 pixels per plot, the spatial resolution of the images taken from the ground was of 4608 *×* 3072 pixels per plot. Despite this loss of resolution, aerial indexes performed very similarly or even better than the ground measurements. This may also help to explain why the ground to aerial correlation for the RGB data was higher in CP than in CA, especially if the added background complexity of CA requires higher spatial resolution for accurate quantification. Limited resolution due to the height level can also constrain the performance of NDVI. Our results show how the use of an active sensor (i.e., with its own source of light), such as the GreenSeeker at ground level, helped to improve the correlation with GY in comparison with the NDVI formulation from the aerial images, although this improvement was rather small. This may be due to the improved normalization of the light and other environmental conditions while using field sensors that take some time to cover all of the study plots.

## 5 Conclusions

Conservation agriculture management practices had a positive effect on increasing yields as compared to conventional ploughed system. These results may help support the adoption of CA to combat declining yields that affect SSA agriculture. Henceforth, in order to fully exploit the yield potential, future efforts should focus on the study of the impact of the genotype selection for a particular management system (e.g., Genotype x Environment x Management interaction). The main point of field phenotyping is to understand the genotypic responses and dissect that traits associated with a better performance under CA as a management system. Thus, further work is required before breeding programs invest resources into a whole new management system.

The use of remote sensing technologies, as presented here, would be increasingly useful for large-scale phenotyping studies. The results suggest, even at early crop growth stages, that the different RGB and multispectral indexes have the potential to effectively assess yield differences under CA conditions, even if their performance is lower than under CP conditions. This is assumed to be mainly due to the residue cover effect on the measurements; however, applying a soil (and stover) mask to the images could help in overcoming this technical problem, which may be best accomplished by the fusion of high resolution RGB with multispectral and/or thermal data or by employing advanced image segmentation algorithms not explored in this study. Nevertheless, the performance of the RGB indexes in predicting yield was less affected by tillage conditions than the multispectral indexes. The indexes that best correlated with yield were mostly related to the greenness of the canopy vegetation, such as the RGB indexes GA and a*, and the multispectral indexes NDVI and RDVI. Finally, the platform proximity effect on the image resolution did not have a negative impact on the performance of the indexes, reinforcing the usefulness of UAV and its associated image processing for high throughput plant phenotyping studies under field conditions.

## Supplementary Materials

The following are available online at www.mdpi.com/2072-4292/10/2/349/s1, Table S1: Regression coeficients (r) and P-value from ANOVA for the relationships between the remote sensing indexes measured at ground against the same indexes measured at aerial level within the conservation (CA), conventional (CP) and the combination of both conditions. These indexes are defined in detail in the Material and Methods. GA, Greener Area; GGA, Greener Green Area; NDVI, Normalized Difference Vegetation Index.

## Abbreviations

The following abbreviations are used in this manuscript:

SSA: Sub-Saharan Africa
RGB: Red-Blue-Green
CA: conservation agriculture
CP: conventional ploughed
NDVI: Normalized Difference Vegetation Index
UAV: unmanned aerial vehicle
GY: grain yield
HIS: Hue-Intensity-Saturation
GA: Green Area
GGA: Greener Area
CSI: Crop Senescense Index
CIMMYT: International Maize and Wheat Improvement Center m.a.s.l. meters above sea level
CP-OES: Inductively Coupled Plasma Optical Emission Spectroscopy
LCC: leaf chlorophyll content
PRI: Photochemical Reflectance Index
SAVI: Soil Adjusted Vegetation Index
MCARI: Modified Chlorophyll Absorption Ratio Index
WBI: Water Band Index
RDVI: Renormalized Difference Vegetation Index
EVI: Enhanced Vegetation Index
ARI2: Anthocyanin Reflectance Index 2
CRI2: Carotenoid Reflectance Index 2
TCARI: Transformed Chlorophyll Absorption in Reflectance Index
OSAVI: Optimized Soil-Adjusted Vegetation Index
ΔF/Fm’: Effective fluorescence quantum yield NIR near-infrared.
