# Supplementary material for: Phenotyping Conservation Agriculture Management Effects on Ground and Aerial Remote Sensing Assessments of Maize Hybrids Performance in Zimbabwe
Source: Remote Sens (Basel). 2018 Feb 24;10(2):349. doi: 10.3390/rs10020349 (PMC7340492; doi:10.3390/rs10020349)
Supplement: Supplementary file 1 [file RS-2019-RS10020349-s1.pdf]

Supplementary Table S1. Regression coeficients (r) and *P*-value from ANOVA for the relationships between the remote sensing indexes measured at ground against the same indexes measured at aerial level within the conservation (CA), conventional (CP) and the combination of both conditions. These indexes are defined in detail in the Material and Methods. GA, Greener Area; GGA, Greener Green Area; NDVI, Normalized Difference Vegetation Index.

	**All conditions**			**Conservation**			**Conventional**		
	**r**		**p-value**	**r**		**p-value**	**r**		**p-value**
Intensity	-0.213		0.000 ***	0.025		0.000 ***	-0.545	***	0.000 ***
Hue	0.835	***	0.000 ***	0.713	***	0.000 ***	0.880	***	0.000 ***
Saturation	0.718	***	0.315	0.098		0.305	0.421	**	0.192
Lightness	-0.309	**	0.000 ***	-0.001		0.000 ***	-0.646	***	0.000 ***
a	0.881	***	0.000 ***	0.740	***	0.000 ***	0.902	***	0.000 ***
b	0.577	***	0.000 ***	0.261		0.000 ***	0.179		0.000 ***
u	0.889	***	0.000 ***	0.703	***	0.000 ***	0.877	***	0.000 ***
v	0.305	**	0.000 ***	0.263		0.000 ***	-0.254		0.000 ***
GA	0.864	***	0.000 ***	0.754	***	0.000 ***	0.911	***	0.042 *
GGA	0.880	***	0.000 ***	0.790	***	0.000 ***	0.922	***	0.000 ***
NDVI plot	0.796	***	0.000 ***	0.654	***	0.000 ***	0.833	***	0.000 ***
NDVI veg	0.835	***	0.000 ***	0.746	***	0.000 ***	0.841	***	0.000 ***
